# 

**Published:** 1849-09

**Authors:** 


### CONTENTS:

### PART I.—ORIGINAL COMMUNICATIONS.

PAGE.
ART. 1.—Masked or Disguised Intermittents—By T. BULLARD, M. D. - - 197
ART. 2.—Remarks on Convulsions of Children, as they occur during paroxysms of Intermittent Fever—By P. A. ALLAIRE, M. D. - - - 206
ART. 3.—Case of Encephalitis, with Ossific Deposites in the Membranes of the Brain—By THOMAS HALL, M. D., M. R. C. S. - - - 212
ART. 4.—Inquiry into Prof. Herrick's Theory of Cholera.—By HENRY RITCHIE, M. D. - - - - - - - - - - - 220
ART. 5.—Suit for Malpractice, - - - - - - - - - - 227
ART. 6.—Cholera—its Cause, Pathology aud Treatment—By E. McARTHUR, M. D. - - - - - - - - - - 230
ART. 7.—Ossification of the Superior Lobe of the Right Lung—By Drs. WILLIAMS & JOHNSON, - - - - - - - - - - 238
ART. 8.—Transactions of the Rock-River Medical Society, - - - 242
ART. 9.—Etherial Solution of Gun Cotton in the treatment of Nævus—By DANIEL BRAINARD, M. D. &c. - - - - - - - - 244
ART 10.—Observations on the spread of Asiatic Cholera, and its Communicable Nature—By JOHN EVANS, M. D. &c. - - - - - - 245
ART. 11.—On the use of Chloroform in Neuralgia—By DANIEL BRAINARD, M. D. &c. - - - - - - - - - - - 286

### PART II.—REVIEWS AND NOTICES OF NEW WORKS.

ART. 1.—Etherization in Childbirth—By Prof. WALTER CHANNING, M. D. &c. - - - - - - - - - - - - 289
ART. 2.—Chloroform and Chloric Ether—By Prof. J. C. WARREN, M. D. &c. - - - - - - - - - - 289
ART. 3.—Etherization in Surgery, with Surgical Remarks—By Prof. JNO. C. WAAREN, M. D. &c. - - - - - - - 290
ART. 4.—Treatise on the Diseases of Old Age—By GEORGE E. DAY, M. D. &c. - - - - - - - - - - 291
ART. 5.—Parturition and the Principles and Practice of Obstetrics, By W. TYLER SMITH, M. D. &c. - - - - - - - 292
ART. 6.—On the Maternal Management of Children in Health and Disease—By THOS. BULL, M. D. &c. - - - - - - 292

### PART III.—EDITORIAL.

ART. 1.—State Medical Associations, - - - - - - 294
ART. 2.—Obituary, - - - - - - - - - 296
ART. 3.—Miscellaneous Medical Intelligence, - - - - - 297

